# Fecal and blood microbiota profiles and presence of nonalcoholic fatty liver disease in obese versus lean subjects

**DOI:** 10.1371/journal.pone.0213692

**Published:** 2019-03-14

**Authors:** Yeojun Yun, Han-Na Kim, Eun-ju Lee, Seungho Ryu, Yoosoo Chang, Hocheol Shin, Hyung-Lae Kim, Tae Hun Kim, Kwon Yoo, Hwi Young Kim

**Affiliations:** 1 Department of Biochemistry, College of Medicine, Ewha Womans University, Seoul, Republic of Korea; 2 Medical Research Institute, Kangbuk Samsung Hospital, Sungkyunkwan University School of Medicine, Seoul, Republic of Korea; 3 Center for Cohort Studies, Total Healthcare Center, Kangbuk Samsung Hospital, Sungkyunkwan University School of Medicine, Seoul, Republic of Korea; 4 Department of Occupational and Environmental Medicine, Kangbuk Samsung Hospital, Sungkyunkwan University School of Medicine, Seoul, Republic of Korea; 5 Department of Family Medicine, Kangbuk Samsung Hospital, Sungkyunkwan University School of Medicine, Seoul, Republic of Korea; 6 Department of Internal Medicine, College of Medicine, Ewha Womans University, Seoul, Republic of Korea; Kaohsiung Medical University Chung Ho Memorial Hospital, TAIWAN

## Abstract

Pathophysiological background in different phenotypes of nonalcoholic fatty liver disease (NAFLD) remains to be elucidated. The aim was to investigate the association between fecal and blood microbiota profiles and the presence of NAFLD in obese versus lean subjects. Demographic and clinical data were reviewed in 268 health checkup examinees, whose fecal and blood samples were available for microbiota analysis. NAFLD was diagnosed with ultrasonography, and subjects with NAFLD were further categorized as obese (body mass index (BMI) ≥25) or lean (BMI <25). Fecal and blood microbiota communities were analyzed by sequencing of the V3-V4 domains of the 16S rRNA genes. Correlation between microbiota taxa and NAFLD was assessed using zero-inflated Gaussian mixture models, with adjustment of age, sex, and BMI, and Bonferroni correction. The NAFLD group (n = 76) showed a distinct bacterial community with a lower biodiversity and a far distant phylotype compared with the control group (n = 192). In the gut microbiota, the decrease in Desulfovibrionaceae was associated with NAFLD in the lean NAFLD group (log2 coefficient (coeff.) = -2.107, *P* = 1.60E-18), but not in the obese NAFLD group (log2 coeff. = 1.440, *P* = 1.36E-04). In the blood microbiota, Succinivibrionaceae showed opposite correlations in the lean (log2 coeff. = -1.349, *P* = 5.34E-06) and obese NAFLD groups (log2 coeff. = 2.215, *P* = 0.003). Notably, Leuconostocaceae was associated with the obese NAFLD in the gut (log2 coeff. = -1.168, *P* = 0.041) and blood (log2 coeff. = -2.250, *P* = 1.28E-10). In conclusion, fecal and blood microbiota profiles showed different patterns between subjects with obese and lean NAFLD, which might be potential biomarkers to discriminate diverse phenotypes of NAFLD.

## Introduction

Nonalcoholic fatty liver disease (NAFLD) is the most common cause of chronic liver diseases.[1] Patients with NAFLD have increased risk of developing cirrhosis or hepatocellular carcinoma, as well as cardiovascular events, malignancies other than hepatocellular carcinoma, and increased mortality.[2, 3] Obesity is a well-documented risk factor for the development of NAFLD.[4] However, the relationship between obesity and NAFLD appears more complicated, considering the absence of NAFLD in obese subjects without any metabolic abnormalities and the presence of NAFLD in lean (body mass index (BMI) <25 kg/m^2^) individuals with metabolic abnormalities such as insulin resistance.[5] Although the prevalence of lean NAFLD shows ethnic preponderance, particularly Asians, it was also found in approximately 10% of Western population.[6–8] However, the pathogenetic differences between phenotypes of NAFLD remain to be elucidated.

The microbiota found in the human body comprise trillions of microorganisms, with the majority colonizing the gut.[9] Gut microbiota appear to be one of the key regulators in the pathogenesis of obesity, diabetes, and metabolic syndrome.[10–12] Recent studies have suggested that gut microbiota are involved in the pathogenesis of NAFLD.[13] For example, gut-derived endogenous alcohol was suggested to play a role in the pathogenesis of nonalcoholic steatohepatitis.[14] In addition, shifts in the composition of gut microbiota seemed relevant in NAFLD, such as decrease in some members of Firmicutes,[15] or abundance of Bacteroidetes in nonalcoholic steatohepatitis and *Ruminococcus* in significant fibrosis.[16] However, these studies mostly focused on obese subjects. Because BMI may be one of the major determinants of compositional changes in gut microbiome,[17] microbial characteristics could be different among NAFLD patients with different body habitus. A recent study has reported Firmicutes-poor microbiota along with marked lower overall microbial richness in nonobese NAFLD compared with nonobese control.[18] However, studies linking gut dysbiosis and phenotypic variations of NAFLD in terms of body habitus are scarce.

A recent pilot study demonstrated that changes in blood microbiota are associated with liver fibrosis in obese patients.[19] The liver has a unique vasculature; it receives the majority of its blood supply from the intestine through the portal vein. Thus, disturbances in the intestinal immune system could increase intestinal permeability and bacterial translocation, triggering various pathological sequences including obesity, metabolic and liver diseases.[20] Recently, the predictive role of blood microbiota has been reported in metabolic diseases.[21, 22] Although studies on blood microbiota attract attention with anticipation of their use as potential noninvasive biomarkers, data on the relationship between gut and blood microbiota and the presence of NAFLD in subjects with different body habitus are insufficient. Thus, we aimed to investigate fecal and blood microbiota profiles in obese versus lean subjects with or without NAFLD.

## Materials and methods

### Study subjects

Health checkup examinees were screened for the eligibility for this study between June and September 2014 at Kangbuk Samsung Hospital Total Healthcare Screening Centers in Seoul, South Korea.[23] The affordable number of participants for analysis within the study budget was less than 300. During the screening period, a total of 296 subjects were found to be eligible showing no evidence of other liver diseases (i.e., positive serology for viral hepatitis B or C, significant alcohol intake (daily alcohol consumption ≥ 30 g [male] or 20 g [female]), other metabolic or hereditary liver diseases, or use of medications such as amiodarone, tamoxifen, methotrexate, or corticosteroids). They gave written informed consents and agreed to provide samples for blood and fecal microbiota analysis. Among these, 268 subjects were finally enrolled in the present study by excluding 28 because of their previous use of antibiotics, probiotics, or cholesterol-lowering medications (n = 22), and the presence of diabetes mellitus (n = 6). Of these 268 participants, NAFLD was diagnosed in 76 subjects based on the presence of ultrasonographic findings suggestive of fatty liver as described below. Because all study participants had no evidence of liver diseases of other etiologies such as alcoholic or viral as described earlier, incident cases with fatty liver were regarded as NAFLD. The control group comprised 192 subjects without any evidence of NAFLD or other liver diseases.

This study was approved by the Institutional Review Board of Kangbuk Samsung Hospital (KBSMC 2013-01-245-008, registered December 23, 2013). All study participants gave their written informed consent to participate in the study. The present study was conducted according to the ethical guidelines of the World Medical Association Declaration of Helsinki.

### Clinical, laboratory, and radiologic assessments

Height and weight were measured by trained nurses with the participants wearing a lightweight hospital gown without shoes. Briefly, height was measured to the nearest 0.1 cm using a stadiometer with the participants standing barefoot. Weight was measured to the nearest 0.1 kg on a bioimpedance analyzer (InBody 3.0 and InBody 720, Biospace Co., Seoul, Korea). BMI was calculated as weight in kilograms divided by height in meters squared. Study subjects were categorized according to their BMI based on the criteria established for Asian populations: underweight, BMI <18.5 kg/m^2^; normal weight, BMI 18.5–23 kg/m^2^; overweight, BMI 23–25 kg/m^2^; and obese, BMI ≥25 kg/m^2^.[24] Insulin resistance was assessed with the homeostasis model assessment of insulin resistance equation, as follows: fasting blood insulin (μU/ml)×fasting blood glucose (mmol/l)/22.5.[25] An ultrasonographic diagnosis of fatty liver was defined as the presence of a diffuse increase in the echogenicity of the liver parenchyma compared with the kidney or spleen.[26, 27] The intra- and inter-observer reliability for the diagnosis of fatty liver was adequately high (kappa statistics of 0.94 and 0.74, respectively).[28]

### DNA extraction and sequence data generation

Fecal samples were immediately frozen after collection. Buffy coat consisting mainly of leukocytes was used for blood samples. 16S rRNA genes were extracted and amplified from specimens using the MO-BIO PowerSoil DNA Isolation Kit (MO-BIO Laboratories, Carlsbad, CA) according to the manufacturer’s instructions. Amplification and sequencing were performed in the same batch as previously described for analysis of bacterial communities. The genomic DNA was amplified using fusion primers targeting 16S V3-V4 rRNA gene with indexing barcodes. All samples were pooled for sequencing on the Illumina Miseq platform according to the manufacturer’s specifications.[29]

### Sequence analysis

Quality filtering, chimera removal, and de novo operational taxonomic unit (OTU) clustering were carried out using the UPARSE pipeline,[30] which identifies highly accurate OTU from amplicon sequencing data. The reads were dereplicated, sorted, and clustered into candidate OTU with removing chimeric OTU. Taxonomic assignment for OTU was annotated by RDP reference (version 16) with an identity threshold of 97% using UTAX command in the UPARSE pipeline. OTU table with taxonomic assignments was transformed to “biom” format for the compatibility of QIIME software (version 1.9; http://qiime.org).[31] Finally, 5,668,793 reads/227 OTUs with a mean of 21,152 (SD = 12,674) sequences per fecal sample and 9,786,870 reads/4,066 OTUs with a mean of 36,518 (SD = 24,966) sequences per blood sample were included for the QIIME analysis. Alpha diversity was calculated using chao1 and phylogenetic diversity (PD) by QIIME, which significant difference between case/control was calculated with 999 Monte Carlo permutation and Bonferroni multiple correction. Beta diversity on Cumulative Sum Scaling (CSS) normalized OTU tables by QIIME was performed using the weighted UniFrac distance metrics based on the phylogenetic distance comparison between communities showing principal coordinate analysis plots.[32] Permutational ANOVA for distance matrix was calculated with 999 Monte Carlo permutation and Bonferroni multiple correction.

### Statistical analysis

The zero-inflated Gaussian mixture (fitZIG) model of metagenomeSeq package version 1.14.2 [32] was used for correlation analysis between CSS normalized count data (as dependent variables) and control versus NAFLD (as independent categorical variables). Age, sex, and BMI covariates were adjusted for regression analysis. Each taxa level that was abundant (>50 normalized counts per sample) and prevalent (present in 10% of samples) in each analysis set was applied to the zero-inflated Gaussian mixture model with Bonferroni multiple correction (an adjusted *P* value <0.05 is significant). This analysis was performed using R software package version 3.2.3 (R Foundation for Statistical Computing, Vienna, Austria).

## Results

### Clinical characteristics

Table 1 summarizes the baseline characteristics of the entire study subjects (n = 268). Subjects with NAFLD (n = 76, 28.4%) showed significantly higher BMI, blood pressure, and metabolic and liver-related laboratory values than those without NAFLD (i.e., control, n = 192).(all *P*-values <0.05) Among lean subjects (BMI <25 kg/m^2^, n = 195), subjects with NAFLD (i.e., “lean NAFLD”; n = 27, 13.8%) also demonstrated significantly higher biometric and laboratory values, except for hemoglobin A1c and aspartate aminotransferase, than lean controls (Table 2). On the contrary, obese subjects (n = 73) showed less distinctive baseline characteristics between those with (i.e., “obese NAFLD”; n = 49, 67.1%) and without NAFLD, particularly without significant difference in age, sex, blood pressure, lipid profiles, and renal function.

**Table 1:** Baseline characteristics of all study participants.

Variable	All (n = 268)	NAFLD (n = 76)	Control (n = 192)	*P*
Age (years)	43.6±8.2	45.3±8.2	42.9±8.2	0.030
Male gender	138 (51.5)	55 (72.4)	83 (43.2)	<0.001
BMI	23.2±2.9	25.7±2.6	22.2±2.4	<0.001
Waist circumference (cm)	80.7±8.7	88.5±6.8	77.7±7.4	<0.001
Glucose (mg/dL)	91.6±7.7	96.0±8.8	89.9±6.5	<0.001
Triglyceride (mg/dL)	100.1±74.9	150.6±92.6	93.0±83.3	<0.001
Total cholesterol (mg/dL)	198.4±32.5	206.9±36.3	195.1±30.4	0.007
HDL cholesterol (mg/dL)	58.3±14.4	49.6±11.5	61.8±14.1	<0.001
LDL cholesterol (mg/dL)	118.5±31.5	128.9±36.4	114.4±28.4	0.001
Systolic blood pressure (mmHg)	106±12	112.0±9.8	103.9±12.0	<0.001
Diastolic blood pressure (mmHg)	69±9	72.6±8.4	66.9±8.8	<0.001
HOMA-IR	1.20±0.78	1.8±1.0	0.95±0.5	<0.001
Insulin (μU/ml)	5.17±3.1	7.49±3.82	4.25±2.08	<0.001
Hemoglobin A1c (%)	5.5±0.2	5.56±0.27	5.46±0.22	0.001
AST (IU/L)	20.0±6.2	22.1±7.3	19.1±5.5	0.002
ALT (IU/L)	18.5±11.6	24.5±12.9	20.9±17.9	<0.001
GGT (IU/L)	24.4±20.0	33.1±22.2	20.9±18.0	<0.001
BUN (mg/dL)	13.7±3.1	13.8±3.3	13.7±3.1	0.843
Creatinine (mg/dL)	0.86±0.18	0.94±0.17	0.82±0.18	<0.001
TyG index	8.4±0.6	8.75±0.53	8.27±0.51	<0.001

The values are expressed as the mean ± standard deviation or frequency (percentage). Abbreviations: NAFLD, nonalcoholic fatty liver disease; BMI, body mass index; HDL, high density lipoprotein; LDL, low-density lipoprotein; HOMA-IR, homeostasis model assessment of insulin resistance; AST, aspartate aminotransferase; ALT, alanine aminotransferase; GGT, gamma-glutamyl transferase; BUN, blood urea nitrogen; TyG, triglyceride-glucose.

**Table 2:** Demographic and clinical characteristics of subjects with NAFLD vs. control according to their body habitus.

Variable	Lean NAFLD (n = 27)	Lean control (n = 168)	*P*	Obese NAFLD (n = 49)	Obese control (n = 24)	*P*
Age (years)	46.7±8.3	42.6±8.2	0.013	44.6±8.1	45.5±9.6	0.666
Male gender	18 (66.7)	66 (39.3)	0.008	37 (75.5)	17 (70.8)	0.669
BMI	22.8±2.6	21.8±1.8	<0.001	27.3±1.6	26.2±1.1	0.001
Waist circumference (cm)	81.9±4.9	76.8±6.2	<0.001	92.0±4.7	87.7±4.1	<0.001
Glucose (mg/dL)	95.1±9.4	89.9±6.7	0.007	96.5±8.5	91.5±5.1	0.003
Triglyceride (mg/dL)	111.8±52.6	92.1±46.1	0.031	172.0±102.9	165.3±162.9	0.830
Total cholesterol (mg/dL)	205.6±34.1	194.2±30.3	0.068	207.6±37.8	202.4±33.4	0.564
HDL cholesterol (mg/dL)	54.1±12.9	62.2±13.1	0.001	47.2±9.9	52.3±14.8	0.134
LDL cholesterol (mg/dL)	130.0±28.6	114.1±28.7	0.005	128.2±40.3	121.3±28.2	0.454
Systolic blood pressure (mmHg)	108.3±10.1	103.3±11.3	0.020	114.0±9.1	111.2±13.8	0.370
Diastolic blood pressure (mmHg)	71.0±9.4	66.5±8.4	0.008	73.5±7.7	71.8±9.4	0.398
HOMA	1.42±0.89	0.95±0.5	0.009	2.02±1.01	1.13±0.46	<0.001
Insulin (μU/ml)	5.93±3.33	4.22±2.08	0.011	8.35±3.83	4.98±1.91	<0.001
Hemoglobin A1c (%)	5.51±0.27	5.45±0.22	0.155	5.59±0.26	5.54±0.22	0.422
AST (IU/L)	20.2±4.8	19.2±5.8	0.330	23.1±8.2	19.3±3.5	0.006
ALT (IU/L)	20.9±10.1	16.0±10.8	0.021	26.6±13.8	18.3±7.8	0.002
GGT (IU/L)	29.1±20.0	20.2±17.9	0.013	35.2±23.2	28.4±19.3	0.214
BUN (mg/dL)	14.6±2.7	13.6±3.1	0.154	13.4±3.5	14.1±2.7	0.360
Creatinine (mg/dL)	0.90±0.18	0.81±0.18	0.009	0.97±0.17	0.90±0.16	0.144

The values are expressed as the mean ± standard deviation or frequency (percentage). Abbreviations: NAFLD, nonalcoholic fatty liver disease; BMI, body mass index; HDL, high density lipoprotein; LDL, low-density lipoprotein; HOMA-IR, homeostasis model assessment of insulin resistance; AST, aspartate aminotransferase; ALT, alanine aminotransferase; GGT, gamma-glutamyl transferase; BUN, blood urea nitrogen; TyG, triglyceride-glucose.

### Microbial diversity of fecal and blood microbiota in the NAFLD group

Alpha diversity measures diversity within a community. Different metrics have been devised to measure alpha diversity with emphasis on the different aspects of the community structure. In Table 3, overall blood microbiota had higher richness (Chao1) but less PD than fecal microbiota. In fecal data, the NAFLD group showed slightly lower biodiversity than the control group, but it was statistically significant only in fecal microbial PD of total NAFLD (Bonferroni corrected *P* = 0.011). The biodiversity of the NAFLD group also showed lower tendencies than that of the control group in blood microbiota as well.

**Table 3:** Comparison of alpha diversity index between control and NAFLD groups using fecal and blood microbiota OTU table.

		Chao1		PD	
	Total	Fecal	Blood	Fecal	Blood
Overall	Control	113.5 ± 24.15	130.0 ± 88.05	18.88 ± 3.08	8.93 ± 6.18
	NAFLD	107.0 ± 27.01	124.5 ± 92.76	17.92 ± 3.40	8.24 ± 5.84
	*P*	0.097	0.618	0.011	0.400
BMI <25	Control	112.8 ± 23.98	129.3 ± 91.05	18.84 ± 3.03	8.92 ± 6.37
	NAFLD	108.4 ± 26.67	128.3 ± 131.2	18.19 ± 3.15	8.66 ± 9.20
	*P*	0.681	0.841	0.165	0.95
BMI ≥25	Control	114.09 ± 23.58	129.7 ± 64.16	19.28 ± 3.61	8.93 ± 5.49
	NAFLD	106.2 ± 27.44	111.3 ± 55.14	17.78 ± 3.56	7.73 ± 2.56
	*P*	1.0	0.098	1.0	0.201

Data are presented as mean ± SD. Abbreviations: NAFLD, nonalcoholic fatty liver disease; OTU, operational taxonomic unit; PD, phylogenetic diversity; BMI, body mass index.

**Fig 1:**
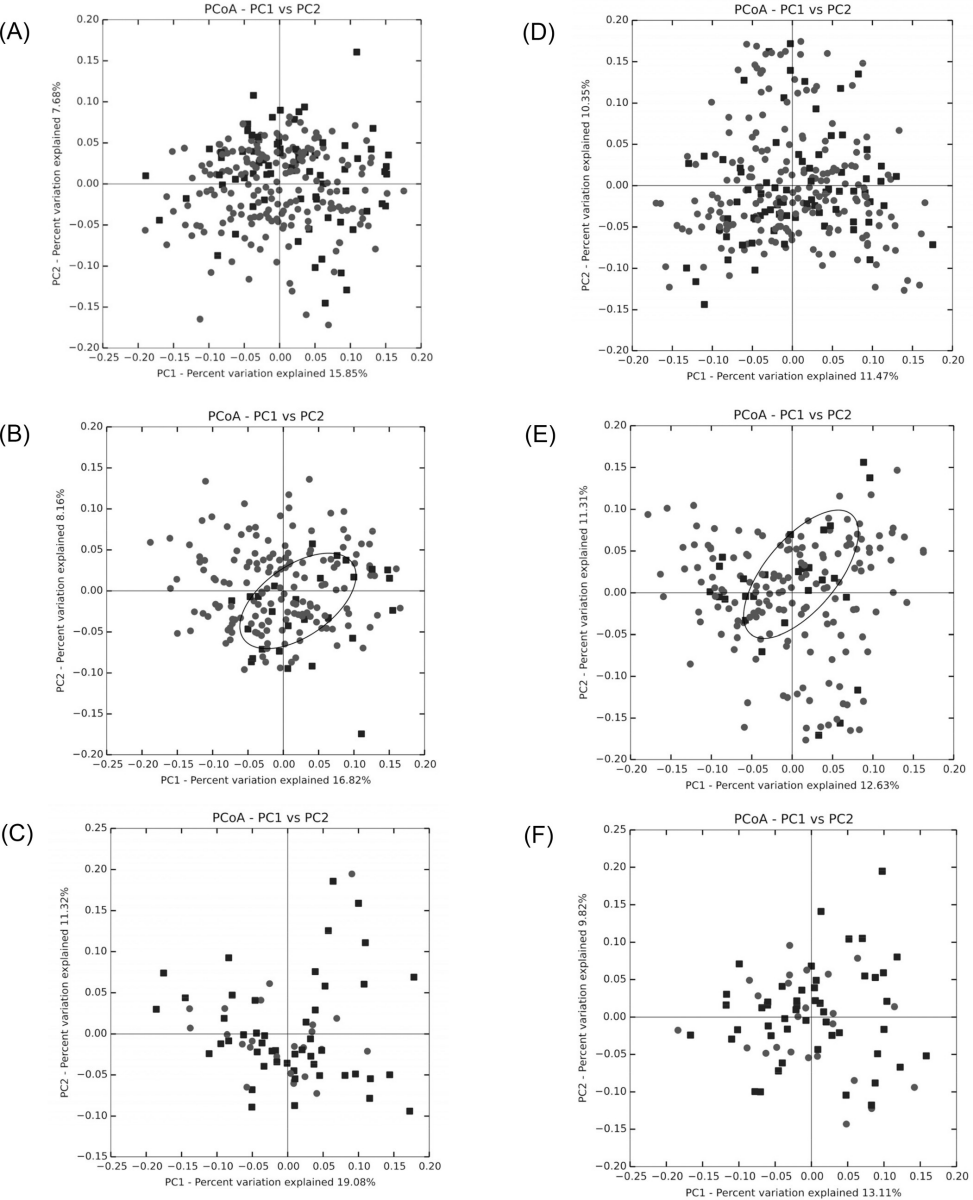
Beta-diversity of principal coordinate analysis plots of fecal and blood microbiota by weighted UniFrac based on the cumulative sum scaling normalized count of operational taxonomic units. NOTE. Nonalcoholic fatty liver disease (black squares) and control (gray circles) groups from total (A [fecal], D [blood]), lean (B [fecal], E [blood]), and obese (C [fecal], F [blood]) samples.

Beta diversity measuring the variations in community membership across the different groups was performed to prove the differentiation between groups using OTU abundance with weighted Unifrac metrics, weighting species abundances with phylogenetic relationships among taxa. In principal coordinate analysis plots of both fecal and blood microbiota, only lean NAFLD showed a clustering of the NAFLD group (Fig 1). When it was examined for statistical significance of the distance metrics, permutational ANOVA of blood microbiota exhibited no significant results. However, permutational ANOVA of fecal microbiota showed highly significant difference between the NAFLD and control groups, except obese NAFLD (Table 4). Therefore, the highly significant difference between the NAFLD and control groups is predominantly due to the difference in lean NAFLD (pseudo-F = 3.021, *P* = 0.001).

**Table 4:** Beta diversity by PERMANOVA (permutaional multivariate analysis of variance) the weighted UniFrac distance between control and NAFLD group.

	PERMANOVA			
	Fecal		Blood	
	pseudo-F	*P*	pseudo-F	*P*
Total NAFLD (n = 76)	2.797	0.002	0.958	0.461
Lean NAFLD (n = 27)	3.021	0.001	0.798	0.752
Obese NAFLD (n = 49)	0.768	0.728	0.862	0.654

Abbreviation: NAFLD, nonalcoholic fatty liver disease.

### Taxonomic comparison in fecal microbiota

To obtain a featured change of microbial components, we used zero-inflated Gaussian mixture based on the normalized count data of an OTU table. Table 5 shows a summary of significant differential bacterial taxa (Bonferroni corrected *P*<0.05) with at least 2 times of coefficient effect, adjusted by age, sex, and BMI. The results of total NAFLD (obese and lean NAFLD) resembled those of lean NAFLD. The decrease in five Clostridia, which belong to Firmicutes, was correlated with lean NAFLD. Two Ruminococcaceae (i.e., *Fastidiosipila* and *Faecalibacterium*) showed same negative patterns in the lean and obese NAFLD as well as in total NAFLD. In particular, *Fastidiosipila* was the only bacterium that showed the same pattern across the three groups. This result indicates the unique bacterial feature for NAFLD regardless of the presence or absence of obesity. However, Desulfovibrionaceae under Deltaproteobacteria showed an opposite trend between lean (negative) and obese NAFLD (positive), which resulted in negative correlation with total NAFLD. The decrease in *Weissella* and its family Leuconostocaceae was associated with only the obese NAFLD group.

**Table 5:** Significant taxa in fecal microbiota related with NAFLD groups by regression analysis.

taxa	Total NAFLD	Lean NAFLD	Obese NAFLD
**Proteobacteria/Gammaproteobacteria/Enterobacteriales/**			
Enterobacteriaceae	-1.239	-1.507	
*Citrobacter*	-1.398	-1.602	
**Proteobacteria/Deltaproteobacteria/Desulfovibrionales/**			
Desulfovibrionaceae	-1.407	-2.107	1.440
*Biophila*	-1.831	-2.451	
**Firmicutes/Bacilli/Lactobacillales/**			
Leuconostocaceae			-1.168
*Weissella*			-1.245
**Firmicutes/Clostridia/Clostridiales/**			
Ruminococcaceae/*Fastidiosipila*	-1.790	-1.823	-2.001
Ruminococcaceae/*Faecalibacterium*	-1.183	-1.637	
Peptostreptococcaceae/*Filifactor*		-1.518	
Gracilibacteraceae/*Gracilibacter*		-1.168	
Lachnospiraceae/*Roseburia*		-1.120	-0.996
**Firmicutes/Negativicutes/Selenomonadales/**			
Acidaminococcaceae/*Acidaminococcus*	-1.159	-1.706	
**Proteobacteria/Betaproteobacteria/Burkholderiales/**			
Sutterellaceae/*Parasutterella*	-1.192	-1.118	
**Firmicutes/Erysipelotrichia/Erysipelotrichales/**			
Erysipelotrichaceae/*Turicibacter*	-1.078	-1.381	
Erysipelotrichaceae/*Erysipelothrix*	-0.942		-1.306

Data are presented as coefficient values (log2 ratio) driven by zero-inflated Gaussian mixture model (fitZig) using metagenomeSeq package, adjusted by age, sex, and BMI. Data with only significant coefficients (log2 ratio≳1) and results (p-value <0.05 corrected by Bonferroni multiple comparison correction) calculated from 90 genera, 41 families, and 11 phyla are shown. P-value 0.0005, 0.0013, and 0.0045 were applied for genus, family and phylum level respectively. Original p-value were provided in S1 Table.

### Taxonomic comparison in blood microbiota

In contrast to fecal data, blood microbiota of total NAFLD were more shared with obese NAFLD pattern, but the obese NAFLD group showed more unique results (Table 6). Notably, the family Leuconostocaceae was negatively associated with obese NAFLD in blood and fecal microbiota. However, the genus under Leuconostocaceae was *Leuconostoc*, not *Weissella*. Associated bacteria including Deinococcus-Thermus and Deferribacteres phyla were much more varied in the blood than in the feces. The decrease in Deferribacteriales incertae sedis was highly associated with obese NAFLD, which was in contrast to lean NAFLD. Anaerobiospirillum and its family Succinivibrionaceae were negatively associated with lean NAFLD, but very positively associated with obese NAFLD. The decrease in rhizobial Beijerinckiaceae and archeal Methanosarcinaceae was highly correlated with obese NAFLD.

**Table 6:** Significant taxa in blood microbiota related with NAFLD groups by regression analysis.

taxa	Total NAFLD	Lean NAFLD	Obese NAFLD
**Deinococcus-Thermus/**	-0.926		-1.605
Deinococci/Deinociccales/Deinococcaceae	-1.118		-1.771
*Deinococcus*	-1.172		-1.756
**Firmicutes/Bacilli/Lactobacillales/**			
Leuconostocaceae	-0.950		-2.250
*Leuconostoc*	-0.880		-2.253
**Firmicutes/Clostridia/Clostridiales/**			
Clostridiaceae/*Clostridium sensu stricto*	-0.657		-1.562
Clostridiaceae/*Clostridium* IV			-1.088
**Actinobacteria/Actinobacteria/Actinomycetales/**			
Norcadioidaceae	-1.267		-1.267
Norcadioidaceae/*Norcadioides*	-1.194		-0.820
Micrococcaceae			-1.247
Microbacteriaceae/*Chryseoglobus*			-1.562
**Proteobacteria/Alphaproteobacteria/Rhizobiales/**			
Beijerinckiaceae	-1.009		-2.255
*Beijerinckia*			-1.788
**Sphingomonadales/**			
Erythrobacteraceae		1.015	
Rhodobacteraceae			1.828
**Deferribacteres/Deferribacteres/Deferribacteriales/**			
Deferribacteriales incertae sedis	0.607	1.515	-2.001
*Caldithrix*	0.600	1.688	
**Proteobacteria/Gammaproteobacteria/Aeromonadales/**			
Succinivibrionaceae		-1.349	2.215
*Anaerobiospirillum*		-0.989	2.259
**Xanthomonadales/**			
Xanthomonadaceae			-1.229
*Lysobacter*			-1.189
Oceanospirillales/Alcanivoracaceae/*Alcanivorax*		1.104	
Legionellales/Legionellaceae/*Legionella*			-1.032
**Verrucomicrobia/Verrucomicrobiae/Verrucomicrobiales/**			
Rubritaleaceae			-1.398
*Rubritalea*			-1.419
**Euryarchaeota/Methanomicrobia/Methanosarcinales/**			
Methanosarcinaceae			-2.182
**Proteobacteria/Deltaproteobacteria/Desulfobacteriales/**			
Desulfobacteraceae	-0.767		-1.431
**Bacteroidetes/Flavobacteriia/Flavobacteriale/**			
Flavobacteriaceae/*Maribacter*			-1.670
*Actibacter*			-1.472
*Aestuariicola*			1.100
**Bacteroidetes/Bacteroidia/Bacteroidales/**			
Porphyromonadaceae/*Parabacteroides*			-1.699
**Proteobacteria/Betaproteobacteria/Burkholderiales/**			
Comamonadaceae/*Delftia*		1.269	

Data are presented as coefficient values (log2 ratio) driven by zero-inflated Gaussian mixture model (fitZig) using metagenomeSeq package. Data with only significant coefficients (log2 ratio≳1) and results (p-value <0.05 adjusted by Bonferroni multiple comparison correction) calculated from 607 genera, 259 families, and 42 phyla are shown. P-value 8.2*10^5^, 1.9*10^4^, and 0.0012 were applied for genus, family and phylum level respectively. Original p-value were provided in S2 Table.

## Discussion

The results of the present study demonstrated that lean subjects had different characteristics in blood microbiota in terms of the presence of NAFLD. Furthermore, lean subjects with NAFLD showed different features in blood and gut microbiota compared with obese subjects with NAFLD. These data suggest that the distinctive features of blood microbiota might be diagnostic for the presence of NAFLD in lean population, which may be used as a point-of-care test for early detection of lean NAFLD.

The results suggested unique pattern of reduced PD in fecal and blood microbiota in lean subjects with NAFLD. The beta diversity did not distinguish between subjects with NAFLD and controls. By contrast, when the subjects were stratified based on their BMI, fecal microbiota discriminated subjects with NAFLD in the lean subgroup and in the overall subjects. Interestingly, blood microbiota showed reduced richness in bacterial diversity in lean individuals with NAFLD in contrast to lean controls or obese group. However, such diversity was not distinct in fecal microbiota between subjects with or without NAFLD, or between subgroups with or without obesity (Table 3). Lower PD in fecal microbiota in overall subjects with NAFLD was in consistent with recent studies.[18, 33] We observed a markedly distinct microbial community in fecal microbiota of subjects with lean NAFLD but not in blood. This discrepancy in ecological diversity between blood and fecal microbiota might have resulted from their genuine compositional difference due to the presence of intestinal barrier, filtering function of the liver, and the role of immune cells.[34] In addition, blood microbiota may have oral source other than gut-derived bacteria.[35] Excluding the possibilities of confounding effects of oral disease in the study participants might help clarify phylogenetic characteristics more evidently.

The vast majority of human gut microbiota consist of three bacterial phyla, namely, Bacteroidetes, Actinobacteria, and Firmicutes.[36] Dysbiosis between beneficial and pathogenic bacteria may lead to obesity, insulin resistance, and NAFLD.[37] In particular, both Bacteroidetes and Firmicutes (phylum) encode carbohydrate-digesting enzymes metabolizing complex carbohydrates to the short-chain fatty acids. A greater number of these enzymes are encoded by Bacteroidetes than by Firmicutes.[38] Obese subjects have an increased Bacteroidetes/Firmicutes ratio and higher short-chain fatty acids than lean subjects.[39] Previous studies on fecal microbiota reported a higher Bacteroidetes/Firmicutes ratio in NAFLD patients than in control.[14, 15] This finding is in line with our results on fecal microbiota showing many bacteria which belong to Firmicutes were decreased in subjects with NAFLD regardless of body habitus (Table 5). However, the level of lactobacilli (family Leuconostocaceae and Weisella) was lower only in obese subjects with NAFLD. In addition, the quantities of Desulfovibrionaceae were significantly different between obese NAFLD and lean NAFLD. These differences in the composition of gut microbiota suggest different characteristics in terms of gut dysbiosis, body habitus, and phenotypes of NAFLD. Previous studies on the association between gut microbiota and obesity and metabolic diseases reported controversial results on BMI and lactobacilli; a negative[40] and a positive correlation[41, 42] were found between lactobacilli and BMI.

Based on the potential linkage among disturbances in the intestinal immune system, bacterial translocation, and pathological consequences including obesity, metabolic and liver diseases,[20] we aimed to investigate the potential role of blood microbiota in discriminating the phenotypes of NAFLD. In our results, the negative correlation of the presence of NAFLD, particularly obese NAFLD, and lactobacilli Leuconostocaceae was also observed in blood microbiota, as well as in fecal microbiota. However, other bacteria showed mixed features, especially in obese NAFLD; for example, reduced Actinomycetales and Deferribacteriales versus increased Aeromonadales (Table 6). Among these organisms, distinctly different correlations were observed in Deferribacteriales incertae sedis and Aeromonadales between lean NAFLD and obese NAFLD. Previous studies demonstrated that some microbial metabolites, such as lipopolysaccharide, are found in the plasma of obese subjects and correlated with the degree of liver inflammation. This finding suggests the role of a mediator in the development and progression of NAFLD.[43, 44] Lean subjects with NAFLD might have different degrees of liver injury from obese patients, as was shown in a recent study from Hong Kong.[45] In addition, lean NAFLD is associated with decreased likelihood of having insulin resistance and hypercholesterolemia compared with overweight or obese NAFLD in a Western study.[6] Taken together, patients with lean NAFLD may have different pathogenetic mechanisms and clinical characteristics other than BMI from obese NAFLD, which might be linked with different gut and blood microbiota profiles in a complex manner. Direct evidences to the pathogenetic link between blood microbiota and NAFLD phenotypes are scarce. However, increase in Proteobacteria in the blood of the obese NAFLD group was observed, which shares similar gut microbiome characteristics of alcoholic liver diseases and cirrhosis.[46, 47] The opposite results of the correlation between the abovementioned bacteria and phenotypes of NAFLD (i.e., lean versus obese) might reflect the different degrees of bacterial translocation and resultant low-grade inflammatory state as well as the degrees of insulin resistance and NAFLD between lean and obese subjects.[22, 48, 49]

The results of the present study might serve as a microbiota signature to predict NAFLD particularly in lean subjects, before progression of NAFLD to significant fibrosis or cirrhosis. In addition, a point-of-care test based on our blood microbiota characteristics might be anticipated if these results are properly validated in the near future. On the contrary, this study has several limitations when interpreting our results. First, clinical characteristics including risk factors for NAFLD or disease severity of NAFLD of the study subjects who volunteered for health checkup were deemed similar to those of the general population, compared to patients with established NAFLD or nonalcoholic steatohepatitis. In addition, although the characteristics of the study participants harbors concerns of selection bias, the prevalence of overall participants or lean subgroup was similar to those in the literature.[1, 50–53] Second, lack of histological data prevented further analysis on the relationship between gut and blood microbiota features and the severity of liver disease. Third, the small number of NAFLD subjects (n = 49 [obese] and n = 27 [lean], respectively) could have contributed the absence of difference in alpha and phylogenetic diversity of the blood and fecal microbiota. Diagnosis of NAFLD based on ultrasonographic findings might also have influenced on the number of cases due to the limited sensitivity of ultrasound to detect hepatic steatosis.[54] Other diagnostic technologies with higher sensitivity for hepatic steatosis such as magnetic resonance imaging proton density fat fraction and controlled attenuation parameter using transient elastography were not available as baseline health checkup data.[55] Finally, the results need to be validated in other populations with different characteristics such as body habitus or dietary habits. In addition, causative relationship between the distinctive features of microbiota and NAFLD, instead of merely innocent bystander, warrants further investigation.

In conclusion, our study revealed distinctive features of gut and blood microbiota in terms of the presence of lean and obese NAFLD. The predictive role of the microbiota profiles requires further validation in a larger cohort with histological data.

## Supporting information

S1 Table Significant taxa in fecal microbiota related with NAFLD groups. Both unadjusted and adjusted *P*-values are provided. (XLSX)

S2 Table Significant taxa in blood microbiota related with NAFLD groups. Both unadjusted and adjusted *P*-values are provided. (XLSX)
